# Application of knowledge graphs in health management for chronic disease patients: a scoping review

**DOI:** 10.1186/s12911-026-03704-w

**Published:** 2026-07-25

**Authors:** Ziting Xu, Chunhao Dai, Haoyuan Li, Wen Li, Teng Yang, Luyao Yan, Lijuan Yang, Minmin Leng

**Affiliations:** 1 Department of Nursing, Shandong Provincial Hospital, Affiliated to Shandong First Medical University, Jinan, Shandong 250021 China; 2 School of Nursing, Shandong University of Traditional Chinese Medicine, Jinan, Shandong China

**Keywords:** Knowledge graph, Chronic disease, Disease management, Digital health, Scoping review

## Abstract

**Background:**

Chronic diseases impose a significant burden on global health. Knowledge graphs (KGs), which integrate and represent multisource information as interconnected networks, provide a promising approach for enabling personalized and dynamic health management. The aim of this scoping review is to systematically map the current landscape of KG applications in chronic disease health management.

**Methods:**

In accordance with the Arksey and O’Malley framework and the PRISMA-ScR guidelines, a systematic search was conducted in eight databases, namely, the Wanfang Database, CNKI, VIP, SinoMed, PubMed, Embase, Web of Science, and CINAHL, from the establishment of the databases to August 2025. Two researchers independently screened the literature and extracted data on the basis of the inclusion and exclusion criteria.

**Results:**

A total of 15 studies published between 2018 and 2025 were included. In these studies, the KG construction process generally involved five stages: data collection, information extraction, knowledge fusion, graph construction, and visualization. KGs were applied across a range of chronic diseases, including metabolic, cardiovascular, and respiratory diseases, as well as cancers. In terms of application scenarios, self-health management was the most common (9 studies, 60.0%), followed by clinical decision support (5 studies, 33.3%), intelligent question answering (4 studies, 26.7%), and health monitoring/early warning (1 study, 6.7%). With respect to system maturity, 12 studies (80.0%) were classified as prototype systems, 3 studies (20.0%) were classified as pilot implementations, and none were classified as clinically deployed systems. The reported outcomes were mainly technical or feasibility oriented, and 7 studies (46.7%) did not report any explicit evaluation metrics.

**Conclusion:**

Knowledge graphs show promise for integrating heterogeneous health data in chronic disease management. However, research has focused predominantly on system development and proof-of-concept validation, with limited high-quality evidence regarding clinical effectiveness. To support clinically meaningful implementation, in future work, rigorous real-world evaluation should be prioritized and challenges related to data quality, interoperability, and model interpretability should be addressed.

**Supplementary Information:**

The online version contains supplementary material available at https://doi.org/10.1186/s12911-026-03704-w.

## Introduction

Chronic diseases impose a significant burden on global health and lead to substantial morbidity, mortality, and health care costs [1, 2]. Effective management of these conditions—which are often long term and require continuous care—is crucial for improving patient outcomes and quality of life. However, prevailing chronic disease management models frequently encounter obstacles such as fragmented health information, limited health care resources, insufficient patient self-management support, and a lack of personalized intervention strategies [3]. Consequently, novel paradigms that can integrate disparate data sources and provide intelligent, dynamic support for health management are urgently needed.

Knowledge graphs (KGs) represent an emerging technology that provides a promising solution to these challenges [4]. By structuring information into interconnected networks of entities, relationships, and attributes, KGs can semantically integrate heterogeneous data from diverse sources, including clinical guidelines, electronic health records, medical literature, and patient-generated data [5]. In the context of chronic disease management, KGs hold the potential to unify disease knowledge, treatment protocols, care guidelines, and individual patient data, thereby enabling capabilities such as risk prediction, personalized education, clinical decision support, and self-management empowerment.

In recent years, a growing number of studies have explored the use of KGs in health care for various chronic conditions, such as diabetes, hypertension, and cancer. These exploratory efforts have demonstrated the potential of KGs in developing intelligent question-answering systems, providing decision aids, and formulating personalized management plans. Nevertheless, research in this domain remains in its early stages, with the literature being relatively scattered. A systematic synthesis that is focused on the construction methodologies, application scenarios, range of chronic diseases addressed, and implementation outcomes of KGs in chronic disease health management is currently lacking. This gap hinders researchers and practitioners from obtaining a comprehensive overview of the landscape, technological trends, and research priorities of the field. To address this knowledge gap, in this study, a scoping review was conducted [6–10] to systematically map and summarize the current state of the use of KGs in health management for patients with chronic diseases.

## Methods

This scoping review was conducted following the methodological framework developed by Arksey and O’Malley [11] and is reported in accordance with the Preferred Reporting Items for Systematic Reviews and Meta-Analyses extension for Scoping Reviews (PRISMA-ScR) statement. The completed PRISMA-ScR checklist is provided in Supplementary Material 1. The research protocol has been registered in the Open Science Framework (https://doi.org/10.17605/OSF.IO/2KBTZ).

### Identifying the scope and review questions

To clarify the scope of the review, we adopted the Participants, Concept, Context (PCC) framework recommended by the Joanna Briggs Institute (JBI) scoping review guidelines [12]:

Participants (P): Patients diagnosed with any chronic disease(s).

Concept (C): The construction, application, or evaluation of knowledge graph technology in the health management of patients with chronic diseases.

Context (C): The full spectrum of chronic disease health management, including clinical care, community-based health management, and patient self-management [13].

Guided by this framework, in this review, we sought to address the following specific questions:

1. What is the process of constructing knowledge graphs?
2. What are the specific application scenarios for knowledge graphs?
3. For which chronic disease patients have knowledge graphs been applied? What were the intervention outcomes?

### Search strategy

A systematic search was conducted in the Wanfang Database, China National Knowledge Infrastructure (CNKI), VIP Database, SinoMed, PubMed, Embase, Web of Science, and CINAHL. The search combined subject headings and free-text terms and covered the period from the inception of each database to August 2025. The following search terms were used: (“Knowledge Graph” OR “Visualized Knowledge Graph” OR “Knowledge Graph Visualization”) AND (“Chronic Disease” OR “Chronic Illness” OR “Lifestyle Disease” OR “Chronic Condition” OR “Chronic Noncommunicable Disease” OR “Chronic Disease Management”). Reference lists of relevant publications were manually screened to ensure comprehensive coverage. The PubMed search strategy was as follows: (“Chronic Disease”[Mesh] OR Chronic Disease*[Title/Abstract] OR Chronic Illness*[Title/Abstract] OR Chronically Ill[Title/Abstract] OR Chronic Condition*[Title/Abstract] OR Condition, Chronic[Title/Abstract] OR Lifestyle Diseases*[Title/Abstract] OR Chronic Non-communicable Diseases[Title/Abstract] OR Chronic Disease Management[Title/Abstract]) AND (Knowledge Graph[Title/Abstract] OR Visualized Knowledge Graph[Title/Abstract] OR Knowledge graph visualization[Title/Abstract] OR knowledge map[Title/Abstract] OR visual analysis[Title/Abstract]). In this search string, the asterisk * serves as a truncation operator to capture both singular and plural forms (e.g., “Chronic Disease” and “Chronic Diseases”), and the MeSH term “Chronic Disease” [Mesh] ensures the inclusion of indexed records with narrower terms. The full database-specific search strategies are provided in Supplementary Material 2.

### Study selection and eligibility criteria

Study selection was performed independently by two reviewers trained in systematic evidence-based methodology. All the retrieved records were imported into EndNote 21 software for duplicate removal. An initial screening was conducted on the basis of the titles and abstracts, followed by a full-text review of potentially eligible articles. Any disagreements during the selection process were resolved through discussion or arbitration by a third reviewer. The operational screening framework and decision rules are presented in detail in Supplementary Material 3.

#### Inclusion Criteria

1. The study participants were patients diagnosed with at least one chronic disease;
2. The study explicitly involved the construction or application of a knowledge graph;
3. The study outcomes pertained to any aspect of patient health management;
4. The article was an original research article published in Chinese or English.

#### Exclusion criteria

1. The full text was unavailable;
2. The article was a nonoriginal publication such as commentary, editorial, review, or study protocol;
3. The article was a duplicate publication.

### Data extraction and charting

**Fig. 1:**
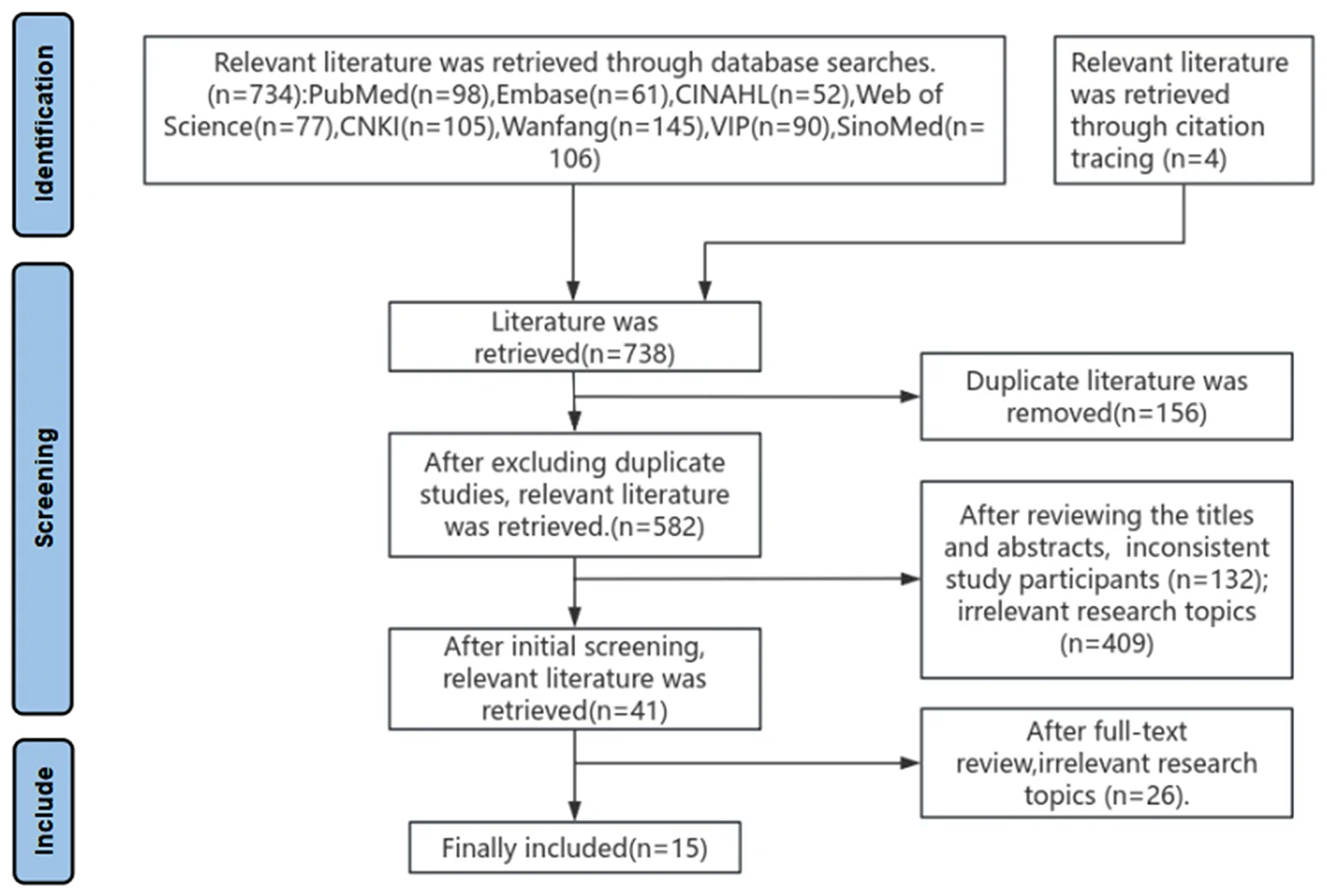
Flow chart of the literature screening

A standardized data extraction form was designed and piloted. The following information was extracted from the included studies: author(s), publication year, country, study population (disease type), sample size, knowledge graph data sources, application scenarios, and reported effects/outcomes. Data extraction was performed by one reviewer and verified by a second reviewer to ensure accuracy. The extracted data were collated and presented through narrative synthesis and summary tables (see Table 1). The complete study selection process is depicted in a PRISMA flow diagram (see Fig. 1).

In addition, during manuscript revision, a qualitative critical appraisal was undertaken to support interpretation of the mapped evidence. Consistent with the scoping review design, this appraisal did not constitute a formal methodological quality or risk-of-bias assessment. Instead, it focused on three narrative dimensions that were particularly relevant to the included knowledge graph studies: system maturity, evaluation methods, and clinical validation status. This supplementary appraisal was used to provide a more cautious and structured interpretation of the heterogeneity of the included studies.

### Qualitative critical appraisal and maturity assessment

Two reviewers (XZT and DCH) independently assessed all the included studies using a predefined appraisal framework that was developed for this review. The systems were classified into three maturity categories.

Prototype systems were defined as systems evaluated primarily through technical validation, retrospective analyses, simulated datasets, or proof-of-concept development without testing that involved real patients, clinicians, or end users. Pilot implementations were defined as systems that underwent small-scale testing in real-world settings that involved intended users or clinical scenarios but had not yet been fully integrated into routine clinical workflows. Clinically deployed systems were defined as systems embedded within routine clinical workflows with sustained operation and systematic monitoring of real-world outcomes.

In addition, information regarding evaluation methods and evidence for clinical validation was extracted and summarized. Any disagreements between reviewers were resolved through discussion, and when consensus could not be reached, a third reviewer (LMM) was consulted. This appraisal was intended to provide a structured narrative assessment of implementation maturity rather than a formal risk-of-bias evaluation.

## Results

### Search results

A total of 738 records were identified through database searching and citation tracing. After duplicate removal, 582 records remained for title and abstract screening. During this stage, 541 records were excluded, including 132 studies with inconsistent study populations and 409 studies with irrelevant research topics. The remaining 41 articles were assessed for full-text eligibility. After full-text review, 26 articles were excluded because they did not meet the inclusion criteria. Consequently, 15 articles were ultimately included: 8 Chinese-language articles [3, 14–20] and 7 English-language articles [21–27]. A flowchart of the literature screening is shown in Fig. 1.

### Basic characteristics for inclusion in the literature study

The publication dates of the included studies ranged from 2018 to 2025. The countries covered included China [3, 14–22, 24, 26], the United States [23, 27], Canada [25], Brazil [23], and Spain [23]. The study subjects included patients with metabolic diseases [3, 18, 21, 26, 27], chronic cardiovascular and cerebrovascular diseases [15, 16, 22], chronic respiratory diseases [19, 27], cancer [24, 25], and other conditions [14, 17, 20, 23, 27]. Sample sizes varied considerably across the studies and ranged from 102 to 6 million cases. Clinical guidelines, expert consensus documents, evidence-based literature, and specialized medical websites served as primary data sources for knowledge graphs. Additionally, in two studies [17, 18], real hospital cases were used as data sources. The primary application scenarios for knowledge graphs included self-health management [3, 16–20, 23, 25, 27], intelligent question-answering [14, 15, 18, 21], and decision support [22–24, 26, 27], with only one study addressing health screening and early warning [27]. The basic characteristics of the included studies are summarized in Table 1.

**Table 1:** Basic characteristics of the included studies (*n* = 15)

Included Study	Country	Conditions Diagnosed in the Study Population	Sample Size	KG Data Sources	Application Scenarios	Reported Outcomes
Qin et al. (2025) [21]	China	Diabetes	11,590	Literature	Intelligent QA	Identification accuracy: 88.55%
Cui et al. (2025) [22]	China	Chronic Cardiovascular Disease	22,475	Guidelines and Expert Consensus	Decision Support	Validated by expert assessment
Correia et al. (2025) [23]	USA, Brazil, Spain	Epilepsy	6,053,578	Guidelines, Literature, Databases, and Websites	Self-management and Decision Support	Usability score: 6.38/7
Li et al. (2024) [24]	China	Gastric Cancer	213	Guidelines and Expert Consensus	Decision Support	Consistency with team > 90%
Chen et al. (2024) [14]	China	Enterostomy Complications	3,730	Textbooks, Literature, and Websites	Intelligent QA	Not reported
Qiao et al. (2024) [19]	China	Atopic Dermatitis & Asthma	631	Guidelines and Websites	Self-management	Not reported
Leng et al. (2024) [17]	China	Dementia	1,012	Clinical Cases	Self-management	Not reported
Hong et al. (2024) [3]	China	Diabetes	147,014	Literature	Self-management	Identification accuracy > 80%
Gao et al. (2023) [16]	China	Hypertension	102	Guidelines and Expert Consensus	Self-management	Not reported
Shao et al. (2023) [18]	China	Diabetes	22,050	Clinical Cases	Intelligent QA and Self-management	Not reported
Wang et al. (2022) [20]	China	Knee Osteoarthritis	206	Guidelines, Literature, and Websites	Self-management	Not reported
Wu et al. (2022) [15]	China	Chronic Cardiovascular Disease	150	Medical Websites	Intelligent QA	Identification accuracy: 95%
Daowd et al. (2021) [25]	Canada	Breast Cancer	3,784	Literature	Self-management	Not reported
Wang et al. (2020) [26]	China	Diabetes	59,936	Literature	Decision Support	Not reported
Gyrard et al. (2018) [27]	USA	Asthma, Obesity, and Parkinson‘s Disease	N/S	Databases	Personalized Management, Decision Support, and Early Warning	Not reported

N/S: not specified; QA: question answering

### Summary statistical analysis of the included studies

To provide a clearer overview of current trends in knowledge graph applications for chronic disease management, we summarized several key characteristics of the included studies on the basis of the data presented in Table 1. In terms of data sources, clinical guidelines or expert consensus documents were used in 6 studies (40.0%), 7 studies (46.7%) relied on medical literature, professional medical websites were incorporated in 5 studies (33.3%), and real-world clinical cases or electronic health record data were used in only 2 studies (13.3%). These findings suggest that current knowledge graph development still depends mainly on publicly available knowledge resources, whereas the use of real-world clinical data remains limited.

With respect to information extraction, automated natural language processing (NLP) methods for entity and relation extraction were used in 7 studies (46.7%), 4 studies (26.7%) relied exclusively on manual extraction, and a hybrid strategy that combines automated extraction with manual verification was adopted in 4 studies (26.7%). In terms of application scenarios, self-health management was the most common use case and appeared in 9 studies (60.0%), followed by clinical decision support in 5 studies (33.3%), intelligent question answering in 4 studies (26.7%), and health monitoring or early warning in only 1 study (6.7%). These findings indicate that applications of KGs have been concentrated mainly in self-management support, whereas warning-oriented applications remain relatively underdeveloped.

The reporting of evaluation metrics was also uneven. Four studies (26.7%) reported recognition accuracy or related technical performance indicators, two studies (13.3%) reported expert assessment or usability outcomes, and one study (6.7%) reported consistency with multidisciplinary team decisions, whereas seven studies (46.7%) did not report any explicit evaluation metrics. Considered together with system maturity, the overall picture remains one of early-stage development: 12 studies (80.0%) were classified as prototype systems, 3 studies (20.0%) were classified as pilot implementations, and no studies were classified as clinically deployed systems.

### System maturity and clinical deployment status

To better characterize the clinical implementation maturity of the included studies, we classified them into three tiers according to their stage of development and deployment: prototype systems, pilot implementations, and clinically deployed systems. Among the 15 included studies, 12 (80.0%) were classified into the prototype systems category. For example, Qin [21] described a diabetes question-answering system that was evaluated in a controlled environment while explicitly noting that real-world deployment remained a direction for future work. Three studies (20.0%) reached the level of pilot implementations. Representative examples include Correia [23], who reported a usability evaluation of the myAURA system for epilepsy management, and Li [24], who examined the consistency of a gastric cancer treatment recommendation system with multidisciplinary team decisions in a clinical setting. No studies met the criteria for clinically deployed systems.

The classification results are summarized in Table 2.

**Table 2:** Classification of included studies by system maturity

Development Stage	Definition	Number of Studies (%)	Included Studies
Prototype systems	Validated using retrospective/simulated data without real-world user testing	12 (80.0%)	Qin et al. (2025) [21]; Cui et al. (2025) [22]; Chen et al. (2024) [14]; Qiao et al. (2024) [19]; Leng et al. (2024) [17]; Hong et al. (2024) [3]; Gao et al. (2023) [16]; Wang et al. (2022) [20]; Wu et al. (2022) [15]; Wang et al. (2020) [26]; Gyrard et al. (2018) [27] and Daowd et al. (2021) [25]
Pilot implementations	Small-scale testing in limited real-world settings	3 (20.0%)	Correia et al. (2025) [23]; Li et al. (2024) [24]; and Shao et al. (2023) [18];
Clinically deployed systems	Integrated into routine workflows with sustained operation	0 (0%)	—

### Medical informatics characteristics of included studies

To further characterize the informatics-related features of the included studies, we summarized ontology or semantic modelling approaches, terminology standards, graph database platforms, interoperability standards, and the use of clinical or EHR-derived data. The results are presented in Table 3.

**Table 3:** Medical informatics characteristics of the included studies

Study	Ontology/Semantic Model	Terminology Standard/Controlled Vocabulary	Graph Database	Interoperability Standard	Clinical Data/EHR Use
Gyrard et al. (2018) [27]	Ontology catalogue reuse	SNOMED-CT, ICD-10, and UMLS	Not reported	Not reported	Proposed integration with EHR and IoT data
Daowd et al. (2021) [25]	Not reported	UMLS (via SemMedDB)	Neo4j	Not reported	Not reported
Wu et al. (2022) [15]	Not reported	Not reported	Neo4j	Not reported	Not reported
Wang et al. (2020) [26]	Not reported	Not reported	Neo4j	Not reported	Not reported
Qin et al. (2025) [21]	Not reported	Not reported	Neo4j	Not reported	Not reported
Cui et al. (2025) [22]	Custom ontology construction	Not reported	Neo4j	Not reported	Not reported
Chen et al. (2024) [14]	Not explicitly reported	Not reported	Neo4j	Not reported	Not reported
Qiao et al. (2024) [19]	Not reported	Not reported	Neo4j	Not reported	Not reported
Leng et al. (2024) [17]	Conceptual knowledge model	Not reported	Neo4j	Not reported	Not reported
Hong et al. (2024) [3]	Not reported	Not reported	Not reported*	Not reported	Not reported
Gao et al. (2023) [16]	Ontology framework reported	Not reported	Neo4j	Not reported	Not reported
Wang et al. (2022) [20]	Not reported	Not reported	Not reported*	Not reported	Not reported
Shao et al. (2023) [18]	RDF-based ontology description	ICD-10	Neo4j	Not reported	EMR data incorporated
Correia et al. (2025) [23]	Not reported	ICD-10	Not reported	Not reported	EHR-derived data used
Li et al. (2024) [24]	Custom ontology construction	Not reported	Neo4j	Not reported	Real-world clinical data used

*The graph database platform was not explicitly reported in the original publication

Abbreviations: EHR, electronic health record; EMR, electronic medical record; IoT, Internet of Things; RDF, resource description framework; SNOMED-CT, Systematized Nomenclature of Medicine Clinical Terms; and UMLS, Unified Medical Language System

None of the included studies explicitly reported the use of HL7 FHIR or DICOM interoperability standards

### Process of knowledge graph construction

**Fig. 2:**
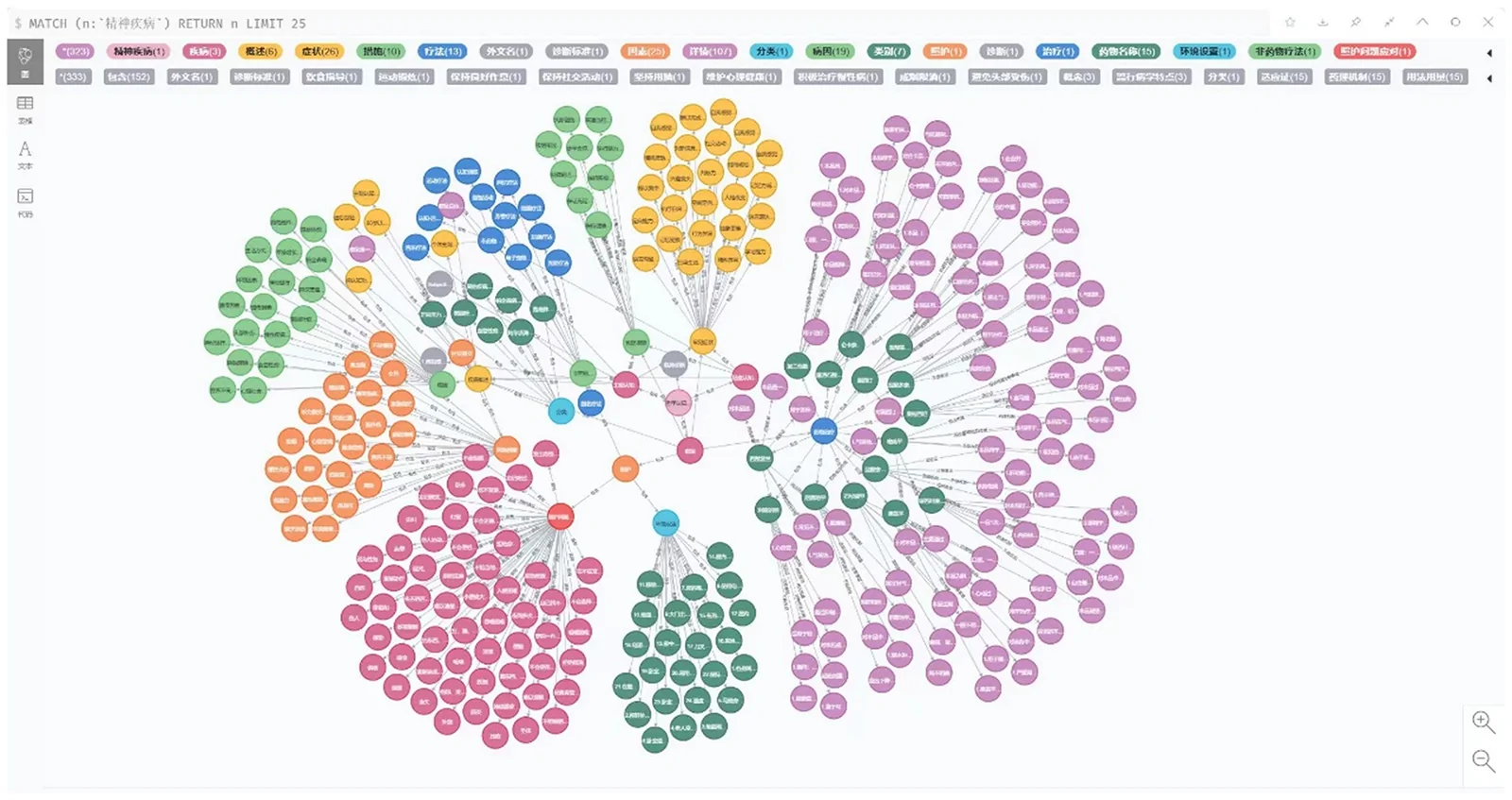
Example of a knowledge graph structure visualized using the Neo4j graph database

In all fifteen included studies, the KG construction process was described in detail and generally adhered to a common workflow that included five sequential stages: data collection, information extraction, knowledge fusion, graph construction, and, finally, visualization and application. The data sources were diverse. In six studies [16, 19, 20, 22–24], primarily clinical guidelines and expert consensus were used, whereas seven studies [3, 14, 20, 21, 23, 25, 26] were based on evidence-based medical literature. Professional medical websites served as foundational corpora in five studies [14, 15, 19, 20, 23]. To increase clinical relevance, in two studies [17, 18], real-world hospital case data were uniquely incorporated into KG development. Information extraction was conducted with the aim of identifying medical entities and their interrelationships. The approaches were categorized into three types. Automated extraction by leveraging NLP algorithms was used in seven studies [3, 15, 21, 23, 24, 26, 27]. In contrast, four studies [14, 17, 19, 20] relied on manual extraction and annotation by researchers with medical expertise. A hybrid strategy that combines both methods was adopted in the remaining four studies [16, 18, 22, 25]. Knowledge fusion involved resolving entity ambiguity and redundancy from multisource data. In eleven studies [3, 15, 16, 18–20, 23–27], terminology standardization was implemented. Entity alignment was achieved in six of these studies [3, 15, 16, 18, 19, 23] by setting similarity thresholds to cluster synonymous terms. This automated process was supplemented with manual verification in three studies [14, 17, 19] to correct omissions. Neo4j was the most frequently reported graph database and was used in the majority of the included studies. An illustrative example of a knowledge graph structure built with Neo4j is shown in Fig. 2.

Visualization strategies varied. Interactive visualizations accessible via web or mobile platforms, which supported operations such as zooming and node focusing, were developed in six studies [14, 17–20, 23]. In four studies [3, 15, 16, 22], only static visualizations were presented, whereas in five studies [24–28], the visualization approach was not explicitly described.

### Application scenarios

On the basis of their functional orientation and target users, the current applications of knowledge graphs in chronic disease management can be broadly categorized into four primary scenarios: intelligent question answering [14, 15, 18, 21], decision support [22–24, 26, 27], self-health management [3, 16–20, 23, 25, 27], and health monitoring and early warning [27]. In intelligent question answering systems, KGs integrated with authoritative, evidence-based knowledge are used to provide instant, accurate responses to patient inquiries via natural language interfaces. For example, one system that was developed for enterostomy complications enables a full cycle of responses from symptom query to nursing guidance through a web-based interface [14]. Decision support applications are designed primarily for clinical settings. By consolidating multidisciplinary guidelines and expert knowledge within a KG, these systems are intended to support diagnosis, treatment planning, and risk assessment. However, the level of supporting evidence varied substantially across the included studies. For example, high consistency of a gastric cancer KG-based clinical decision support system (CDSS) with multidisciplinary team decisions was reported, thus suggesting preliminary clinical applicability [24]. In contrast, other systems were evaluated mainly in controlled or experimental settings. Therefore, the current evidence more strongly supports technical feasibility and preliminary clinical utility than confirmed improvements in real-world decision quality or patient outcomes. Self-health management involves the adoption of a patient-centric approach. KGs combine individual patient characteristics such as age, complications, and treatment duration to generate personalized guidance on diet, exercise, and medication adherence. This guidance supports patients in monitoring key long-term indicators and sustaining self-management behaviours. Health monitoring and early warning systems connect to data streams from sources such as wearable devices. By leveraging the associative reasoning ability of KGs, they identify patterns that are correlated with early disease risk, thereby enabling proactive risk warnings.

### Types of chronic diseases in knowledge graph applications

KGs have been applied to a wide spectrum of chronic diseases, with applications concentrated predominantly in several key categories.

Metabolic diseases, particularly diabetes [3, 18, 21, 26], constitute the most studied category. Research in this category was focused on improving health management through applications in blood glucose control, complication identification, and self-management support. Obesity was also included in this category [27].

Chronic cardiovascular and cerebrovascular diseases, including hypertension [16] and general cardiovascular disease [15, 22], were another area of focus. Related KGs were designed primarily to integrate disease and medication knowledge to support long-term self-management outside hospital settings.

Chronic respiratory diseases, such as asthma and its comorbidities [27], were targeted for management using KGs that emphasized personalized health guidance.

Cancers, including breast [25] and gastric [24] cancer, were explored in a relatively small number of studies. The application focus in this category leaned more towards risk assessment and treatment decision support rather than daily self-management.

Research on other chronic conditions, including enterostomy complications [14], knee osteoarthritis [20], dementia [17], epilepsy [23], and Parkinson’s disease [27], was also reported. Research in these areas typically addressed specific needs, such as complication management, care support, and personalized guidance.

## Discussion

This scoping review provides a systematic mapping of the current landscape of knowledge graph applications in health management for patients with chronic diseases. Our synthesis of 15 included studies elucidates the typical KG construction workflow, summarizes four dominant application scenarios, identifies the spectrum of chronic diseases targeted, and highlights key characteristics and challenges in the current research field.

### Complexity and methodological diversity in knowledge graph construction

Constructing a KG for chronic disease management is a complex, interdisciplinary endeavour that involves integrating clinical expertise with information technology. Our review confirms that the process generally follows the five-stage framework that is common in KG development—data collection, information extraction, knowledge fusion, graph construction, and visualization—yet exhibits specific complexities and diversities in the medical domain.

This complexity stems first from the integration of multisource, heterogeneous data. Building a high-quality KG requires harmonizing authoritative but potentially outdated clinical guidelines [29], real-world clinical cases rich in detail but variable in quality [30], and vast volumes of medical literature, which requires rigorous curation. Although this diversity improves knowledge coverage, it imposes significant demands on data cleaning and standardization. Second, the depth of information extraction directly affects knowledge quality. Although automated extraction using NLP is prevalent, the nuance and complexity of medical language necessitate manual verification to ensure the accuracy of identified entities and relationships. Third, semantic alignment during knowledge fusion is both a technical challenge and a critical step for ensuring logical consistency within the graph. Most of the included studies addressed this through entity alignment on the basis of similarity thresholds, which underscores the importance of terminology standardization.

Methodological diversity is reflected in goal-oriented design. Depending on the specific management needs of different chronic diseases, KGs require tailored adjustments in entity-relationship definitions, attribute granularity, and inference rule design. This diversity suggests that no universal development framework currently exists; successful KG development must be closely coupled with specific clinical scenarios and management objectives.

### Medical informatics considerations: ontology, terminology, and interoperability

Knowledge graphs are fundamentally semantic technologies, and their clinical value depends largely on whether they are supported by standardized ontologies, clinical coding systems, and interoperable data structures. In the studies included in this review, however, reporting of these medical informatics aspects was often limited and uneven. A few studies provide relatively explicit semantic descriptions. For example, Cui [22] clearly described the ontology construction process, distinguished an ontology module from a data module, and reported terminology standardization procedures such as term classification, synonym unification, and normalization of medication expressions. Shao [18] also provided more concrete technical detail and reported the use of the ICD-10 for terminology management and the resource description framework (RDF) for ontology description while incorporating hospital electronic medical records into knowledge graph construction. The most explicit example was provided by Gyrard [27], who described the use of SNOMED-CT, UMLS, and ICD-10, as well as ontology catalogue reuse, and proposed the integration of electronic medical records with Internet of Things (IoT)- and web-derived data. In contrast, most of the remaining studies did not clearly report whether formal ontologies were constructed or reused, and in many cases, only entity–relationship structures were described, without further semantic specification.

A similar pattern was observed in relation to the terminology systems and coding standards. Only a few studies explicitly reported the use of standardized terminology resources or coding systems. Examples include the ICD-10 in Shao [18] and Correia [23]; UMLS through SemMedDB in Daowd [25]; and SNOMED-CT, UMLS, and ICD-10 in Gyrard [27]. Most studies referred more generally to terminology standardization, synonym merging, or concept normalization but did not specify which coding systems were used. This omission is not trivial because the use of standardized vocabularies is central to semantic interoperability and to the reuse of knowledge graph resources across health care settings.

Interoperability with existing health care information systems has been even less consistently reported. Gyrard [27] again provided the clearest example by describing integration across electronic medical records, IoT data, and web-based sources, although without explicit reference to widely used interoperability frameworks such as Health Level 7 Fast Health Care Interoperability Resources (HL7 FHIR). In contrast, most other studies did not clearly describe how their knowledge graphs were connected to electronic health records or other clinical information systems. Qin [21], for instance, explicitly identified integration with clinical decision support systems and electronic health records as a future direction, which suggests that such interoperability had not yet been achieved in that system. More broadly, none of the included studies explicitly reported compliance with HL7 FHIR, DICOM, or other established standards for health care data exchange. These findings suggest that the informatics foundations of current knowledge graph research in chronic disease management remain underreported. Although some studies demonstrated meaningful efforts in ontology design, terminology handling, and semantic structuring, most did not provide sufficient detail to determine whether their systems could be readily reused, mapped, or integrated into broader digital health infrastructures. At present, the evidence more clearly supports the role of knowledge graphs in organizing domain knowledge and structuring terminology than in achieving robust, standards-aware interoperability with routine health care information systems. Future studies would therefore benefit from more explicit reporting of ontology reuse or construction, the coding systems used, and the interoperability frameworks used to support integration with electronic health record systems and other clinical data environments.

### Recent advances: From static knowledge graphs to clinically interoperable intelligent systems

Recent studies have suggested several emerging directions that may help move knowledge graph applications beyond the relatively static prototype systems identified in this review. An area of growing interest is the integration of knowledge graphs with retrieval-augmented generation. Approaches such as graph retrieval-augmented generation (graph RAG) and other graph-enhanced reasoning frameworks aim to combine structured semantic representations with more flexible retrieval and generation capabilities. Early examples include systems developed for medical question answering, agent-oriented knowledge graphs designed to incorporate evolving evidence, and initial validation studies in more complex clinical scenarios [31–33]. Although these studies were not part of the evidence base included here, they reflect a shift towards more adaptive and reasoning-oriented uses of knowledge graphs. Another related development concerns interoperability. Standards such as HL7 FHIR are being increasingly used to support data exchange across health care systems and may provide a practical route for connecting knowledge graphs with electronic health records and other clinical data environments. Previous work has demonstrated the value of FHIR for interoperable patient records, and more recent studies have explored its role in supporting scalable data models and implementation strategies [34, 35]. By comparison, most of the studies included in this review did not report this type of standard-based integration. Even when connections across data sources were described, the use of established interoperability frameworks was generally unclear [27]. This gap helps explain why many knowledge graph systems remain difficult to integrate into routine clinical workflows. More broadly, in recent conceptual work, integrated approaches that combine knowledge graphs with large language models, wearable devices, and interoperable health data infrastructures have begun to be outlined. For example, the “digital-intelligent precision health management” framework proposed by Ma et al. (2026) illustrates how these components might be brought together in the context of chronic disease management [36]. Although these developments fall outside the scope of the current review, they offer a useful indication of how the field may evolve, with increasing emphasis on dynamic data integration, real-time reasoning, and closer alignment with clinical practice.

### Functional value in application scenarios

The four application scenarios synthesized in this review—intelligent question answering, decision support, self-health management, and health monitoring/early warning—closely align with core pain points in contemporary chronic disease management.

Intelligent question answering systems directly address the fragmentation of knowledge and information overload faced by patients and caregivers in home-based management [37]. By providing immediate, authoritative answers, they help correct misconceptions and improve health literacy. Decision support applications assist health care professionals by consolidating and reasoning with multidisciplinary knowledge. These applications have the potential to facilitate more standardized care and reduce practice variation [24]. The self-health management scenario involves a functional transition from “passive information retrieval” to “active interventional guidance”. By generating personalized recommendations on the basis of individual patient profiles, KGs support patient self-management, thereby potentially improving self-efficacy and adherence—a crucial factor in managing conditions that require long-term behavioural changes. Health monitoring and early warning represent more proactive directions. By analysing the associations between real-time data and the knowledge base, KGs may provide potential value for the early identification of risks, thereby potentially contributing to a transition from reactive management towards more preventive approaches [27].

### Qualitative critical appraisal of the included studies

Although scoping reviews do not necessarily require a formal methodological quality or risk-of-bias assessment, because of the heterogeneity of the included studies, some degree of critical interpretation is necessary. Taken together, the current evidence base on knowledge graphs in chronic disease management remains immature in several important respects. Most of the included studies were still situated at the level of prototype development, with 12 of the 15 studies classified as prototype system studies and only 3 reaching the stage of pilot implementation, whereas none could be regarded as clinically deployed system studies. This pattern suggests that the field is still dominated by proof-of-concept work and early technical exploration rather than routine clinical application.

The evaluation of these systems was equally uneven. A few studies reported quantitative indicators such as recognition accuracy or agreement with expert teams, including those by Qin et al. (2025) [21], Hong et al. (2024) [3], Wu et al. (2022) [15], and Li et al. (2024) [24], whereas Cui et al. (2025) [22] relied mainly on expert assessment, and Correia et al. (2025) [23] reported usability outcomes. In contrast, seven studies did not provide any explicit evaluation metrics and instead focused primarily on system construction or possible application scenarios. Even when evaluation was reported, the outcomes were largely limited to technical feasibility, usability, or expert alignment rather than clinically meaningful measures. As a result, comparisons across studies remain difficult, and the overall strength of the evidence is constrained not only by differences in design but also by the inconsistency of the reporting.

A similar limitation is evident in relation to clinical validation. None of the included studies involved the use of randomized or quasiexperimental designs to examine whether knowledge graph systems improved clinical decision-making, workflow efficiency, or patient outcomes, and none reported sustained follow-up after implementation in real-world practice. A few studies, such as those of Correia et al. (2025) [23], Li et al. (2024) [24], and Shao et al. (2023) [18], moved somewhat closer to clinically relevant evaluations by incorporating user feedback, consistency testing, or limited application in practice-related settings, but these efforts were exploratory.

### Characteristics of disease application distribution and clinical suitability

In this review, we reveal that the application of KGs in chronic disease management has formed a core-periphery distribution centred on high-burden conditions while extending to a broader range of diseases. This pattern closely corresponds to the current disease spectrum and real-world burden of chronic diseases in China [18]. For example, owing to the large patient base, long disease course, and clear demands for frequent daily management of diabetes [38], the strengths of KGs in terms of integrated information delivery and personalized recommendation for the management of diabetes are readily demonstrable, which explains the status of diabetes as the most intensively studied area.

Notably, KG applications have also been explored for relatively niche conditions, such as enterostomy complications [14] and dementia [17]. Although the number of studies has been limited, these efforts have targeted core clinical challenges such as complication management and care support, with a pragmatic, problem-driven logic reflected in their design.

Furthermore, the focus of KG applications varies distinctly across disease categories when viewed through the lens of clinical management pathways: they tend to support patient self-management in metabolic diseases, emphasize structured medication knowledge in cardiovascular diseases, and prioritize risk assessment and treatment aid in cancer. These differences are not incidental but are intrinsically linked to the inherent diagnostic, therapeutic, and management logic of each disease category. Metabolic diseases require long-term patient engagement in daily behaviours that directly influence prognosis [39]. Cardiovascular diseases involve complex drug regimens and comorbidities, thus creating a pressing need for precise medication knowledge matching [40]. Cancer care necessitates personalized plans that are based on multidimensional risk factors, thus demanding high expertise in risk stratification and treatment decision-making [41].

This observation underscores a critical implication for future work: the development of disease-specific KGs should be functionally designed around the actual management workflows and pivotal decision points of the target condition rather than by the application of generic templates. Closer alignment with real-world clinical scenarios may help knowledge graphs better address practical needs and realize their potential value. The small number of eligible studies (*n* = 15) and the limited replication across studies for the same disease underscore the early-stage and fragmented nature of this research field.

### Analysis of evidence for clinical decision-making effectiveness

Although several of the included studies suggested that knowledge graphs may contribute to diagnosis, treatment planning, risk assessment, and the provision of evidence-based, personalized guidance, the current evidence remains too limited to support firm conclusions about their effectiveness in improving clinical decision-making or patient outcomes. A closer reading of the literature reveals that what has been evaluated thus far is largely technical feasibility rather than clinically meaningful impact. None of the 15 included studies directly examined whether knowledge graph–based systems improved the quality of clinical decisions, for example, by reducing misdiagnosis, increasing diagnostic accuracy, or improving adherence to guideline-recommended care. Li et al. (2024) [24] reported a high level of consistency with multidisciplinary team decisions (> 90%), but this finding is better understood as preliminary validation against expert judgement than as direct evidence that the system improved real-world decision-making.

A similar gap is evident in relation to workflow efficiency. Although one of the proposed advantages of knowledge graph systems is that they may help organize information and support clinicians more effectively, none of the included studies quantitatively evaluated whether their use reduced clinician time per encounter, lowered the documentation burden, or improved resource utilization in practice. In other words, whether these systems make clinical work more efficient in measurable terms has not yet been shown in the literature. The same limitation applies to patient outcomes. None of the studies reported patient-level outcome measures such as complication rates, hospital readmission, mortality, or quality of life. As a result, there is currently no direct evidence that the use of knowledge graphs leads to better clinical outcomes for patients with chronic diseases. The outcomes reported in the literature remain concentrated mainly on indicators of technical feasibility, such as recognition accuracy, expert assessment, and usability, and several studies did not report explicit evaluation metrics at all. At this stage, therefore, the role of knowledge graphs in chronic disease management is more appropriately understood in terms of technical potential and exploratory utility than as confirmed effectiveness in improving clinical decision-making.

### Challenges in moving towards clinical practice

The literature reviewed here helps explain why knowledge graph applications, despite their promise, have rarely moved beyond technical demonstration. One issue lies in the underlying data. Most systems draw on a mix of guidelines, expert consensus, literature, websites, and, only occasionally, real clinical cases. Although this approach expands coverage, it also introduces uneven terminology, differences in granularity, and variable reliability across sources. In many studies, the processes used for data cleaning, normalization, and alignment were only briefly described or not reported in detail. In the context of chronic disease management, where decisions often depend on comorbidity patterns, medication history, and changes over time, such inconsistencies can directly limit practical use. Similar issues arise at the implementation level. Most studies did not clearly describe how their systems could be connected to electronic health records or other routine clinical systems, and the use of interoperability frameworks was rarely specified. As a result, many knowledge graph applications remain standalone tools rather than part of routine care. Even when technical performance appeared promising, it remained unclear whether clinicians or patients would use these systems in practice. In only a few studies was workflow fit, interpretability, or operational burden considered, thus leaving open questions about whether these tools would save time or add complexity. Another limitation is that most systems were developed on relatively fixed datasets, whereas chronic disease management depends on continuously changing information, including repeated measurements, treatment adjustments, and long-term follow-up. Issues such as scalability, updating, and real-time performance were rarely addressed. Taken together, progress in this area will depend not only on improving algorithms or expanding knowledge bases but also on addressing data quality, interoperability, and implementation in a more systematic way. Without attention to these factors, knowledge graph systems are likely to remain at the level of descriptive or assistive tools rather than becoming part of routine clinical practice.

In future research, greater emphasis should be placed on ontology reuse, semantic interoperability, governance mechanisms, and standards-based integration to support sustainable and clinically scalable knowledge graph systems.

### Limitations

This review has several limitations. First, as a scoping review, the primary aim of this study was to map the current landscape of knowledge graph applications in chronic disease management rather than to conduct a formal methodological quality assessment or risk-of-bias evaluation of the included studies. Accordingly, standardized appraisal tools were not applied, which limits the ability to make systematic judgements regarding internal validity. Second, the clinical implementation maturity is extremely low. According to the three-tier (prototype systems, pilot implementations, and clinically deployed systems) classification framework adopted in this review, among the 15 included studies, 12 (80.0%) involved prototype systems, 3 (20.0%) involved pilot implementations, and none involved clinically deployed systems. This distribution indicates that the vast majority of the studies remained at the proof-of-concept or laboratory stage, without sustained deployment or outcome monitoring in routine clinical workflows. Therefore, the evidence base synthesized in this review reflects primarily early-stage technical development rather than established clinical practice. Third, evidence regarding clinical effectiveness remains limited. Most studies reported only technical feasibility indicators such as recognition accuracy, usability, and expert assessment, whereas patient-level outcomes and workflow-related outcomes were rarely evaluated. Fourth, the reporting of medical informatics aspects was highly inconsistent across the included studies. Although a few of the studies described ontology construction, terminology standardization, or elements of semantic interoperability, most did not clearly report whether standardized clinical coding systems or interoperability frameworks were used, nor did they provide sufficient information on integration with electronic health records. Fifth, knowledge representation was static, and updating mechanisms were lacking. Most knowledge graphs in the included studies were essentially static repositories derived from clinical guidelines and the literature, with little discussion of how they could be kept up to date. Almost none of the studies reported mechanisms for updating graphs as medical evidence evolves or patient data accumulate. Critical issues such as scalability, real-time performance, and incremental learning were rarely addressed, which limits the long-term practical value of these systems in real-world chronic disease management. As a result, assessing the extent to which the reported knowledge graph systems could support semantic interoperability or be transferred across different health care information environments was difficult. Sixth, none of the included studies described how patient data were tracked, anonymized, or protected. This gap is particularly concerning given that real-world clinical cases were used in two studies without reporting any data governance or deidentification procedures. The findings of this review should be interpreted as a mapping of an emerging technical field rather than as confirmation of established clinical effectiveness. Seventh, 13 of the 15 included studies originated from China. This distribution may reflect differences in research priorities, health care systems, and publication practices across regions, which may limit the generalizability of the findings to other informatics and health care contexts. In addition, because the search strategy was designed to identify studies that explicitly described knowledge graph applications in chronic disease management, studies that focused primarily on ontology engineering, semantic modelling, or interoperability infrastructure may not have been fully captured. Therefore, this review may underrepresent research in which formal ontology development and semantic interoperability were emphasized.

### Future directions

Future research will need to move beyond predominantly static, technology-oriented knowledge graph construction towards systems that are more dynamic, interoperable, and embedded in clinical practice. Given that most existing studies remain at the stage of prototype development and preliminary validation, a central priority is to strengthen evaluation in real-world care settings and to determine whether these systems can deliver measurable improvements in decision quality, workflow efficiency, care coordination, and patient outcomes.

Moreover, emerging approaches such as graph RAG and related graph-enhanced reasoning frameworks suggest new possibilities for more adaptive clinical question answering and decision support, and standards-based interoperability through frameworks such as HL7 FHIR may provide a practical route for integration with electronic health records and other routine information systems. Progress in this area will also depend on more transparent ontology design, clearer terminology standardization, closer alignment with clinical workflows, and more consistent reporting of system maturity, evaluation methods, and interoperability mechanisms. In addition, questions of system maintenance and governance, particularly how knowledge graphs are updated as clinical evidence evolves, remain largely unaddressed. Moreover, the motivation and feasibility of deploying multiple coordinated knowledge graphs within the same clinical setting, including the technical architectures (e.g., federated knowledge graphs and knowledge hubs) that would enable such integration, remain unexplored and warrant future investigation.

## Conclusions

For chronic disease management, a continuous, longitudinal model of care that spans prevention, treatment, and long-term support is increasingly being adopted [42]. Within this context, knowledge graphs demonstrate technical promise as tools for integrating heterogeneous health information and supporting multiple functions in chronic disease management. This scoping review maps the current landscape, identifying typical construction workflows, four dominant application scenarios, and a spectrum of targeted chronic diseases. However, research has been situated predominantly at the prototype or proof-of-concept stage, with limited evidence of mature real-world implementation in routine clinical workflows. In future research, rigorous real-world clinical effectiveness evaluations should be prioritized to bridge the evidence gap between “technical feasibility” and “clinical effectiveness”. Additionally, future studies should move beyond the construction of static, guideline-derived knowledge graphs and instead focus on developing dynamic updating mechanisms, integrating real-time clinical data streams, and adopting emerging paradigms such as GraphRAG to generate genuinely novel clinical utility.

## Supplementary Information

Below is the link to the electronic supplementary material.

Supplementary Material 1

Supplementary Material 2

Supplementary Material 3

## Data Availability

All data generated or analysed during this study are included in this published article [and its supplementary information files].
