# Supplementary material for: Application of knowledge graphs in health management for chronic disease patients: a scoping review
Source: BMC Med Inform Decis Mak. 2026 Jul 25;26:401. doi: 10.1186/s12911-026-03704-w (PMC13651244; doi:10.1186/s12911-026-03704-w)
Supplement: Supplementary file 2 [file 12911_2026_3704_MOESM2_ESM.docx]

**Search Strategy**

| 1. Pubmed | |
| --- | --- |
| #1 | Search "Chronic Disease"[Mesh] |
| #2 | Search (Chronic Disease*[Title/Abstract]) OR Chronic Illness*[Title/Abstract] OR Chronically Ill[Title/Abstract] OR Chronic Condition*[Title/Abstract] OR Lifestyle Diseases*[Title/Abstract] OR Chronic Non-communicable Diseases[Title/Abstract] OR Chronic Disease Management[Title/Abstract] |
| #3 | #1 OR #2 |
| #4 | Search ((((Knowledge Graph[Title/Abstract]) OR ((Visualized Knowledge Graph[Title/Abstract]) OR (Knowledge graph visualization[Title/Abstract]))) OR (knowledge map[Title/Abstract])) OR (visual analysis[Title/Abstract])) |
| #5 | #3 AND #4  Items found: 98 |
| 2. Embase | |
| #1 | 'chronic disease'/exp |
| #2 | 'Chronic Disease*':ab,ti OR 'Chronic Illness*':ab,ti OR 'Chronically Ill':ab,ti OR 'Chronic Condition*':ab,ti OR 'Condition, Chronic':ab,ti OR 'Lifestyle Diseases*':ab,ti OR 'Chronic Non-communicable Diseases':ab,ti OR 'Chronic Disease Management':ab,ti |
| #3 | #1 OR #2 |
| #4 | 'knowledge graph'/exp |
| #5 | 'Visualized Knowledge Graph':ab,ti OR 'Knowledge graph visualization':ab,ti OR 'visual analysis':ab,ti |
| #6 | #4 OR #5 |
| #7 | #3 AND #6  Items found: 61 |
| 3. Web of Science | |
| #1 | TS=(Chronic Disease* OR Chronic Illness* OR Chronically Ill OR Chronic Condition* OR Condition, Chronic OR Lifestyle Diseases* OR Chronic Non-communicable Diseases OR Chronic Disease Management) |
| #2 | TS=(Knowledge Graph OR Visualized Knowledge Graph OR Knowledge graph visualization OR knowledge map) |
| #3 | #1 AND #2  Items found: 77 |
| 4. CINAHL | |
| S1 | (MH "Chronic Disease") |
| S2 | TI (chronic disease* or chronic illness* or chronically ill or chronic condition* or lifestyle disease* or chronic non-communicable disease* or chronic disease management) OR AB (chronic disease* or chronic illness* or chronically ill or chronic condition* or lifestyle disease* or chronic non-communicable disease* or chronic disease management) |
| S3 | S1 OR S2 |
| S4 | (MH "Knowledge Graph") |
| S5 | (MH "knowledge map") |
| S6 | (MH "Visualized Knowledge Graph") |
| S7 | (MH "Knowledge graph visualization") |
| S8 | (MH "visual analysis") |
| S9 | S4 OR S5 OR S6 OR S7 OR S8 |
| S10 | S3 AND S9  Items found: 52 |
| 5. CNKI | |
| S1 | SU=('慢性病' + '慢性疾病' + '慢性非传染性疾病' + '生活方式病' + '慢性病管理') |
| S2 | SU=('知识图谱' + '可视化知识图谱' + '知识图谱可视化') |
| S3 | S1 AND S2  Items found: 105 |
| 6. Wanfang | |
| S1 | K=(慢性病 OR 慢性疾病 OR 慢性非传染性疾病 OR 生活方式病 OR 慢性病管理) |
| S2 | K=(知识图谱 OR 可视化知识图谱 OR 知识图谱可视化) |
| S3 | S1 AND S2  Items found: 145 |
| 7.SinoMed | |
| S1 | "慢性病"[常用字段:智能] |
| S2 | (慢性疾病 OR 慢性非传染性疾病 OR 生活方式病 OR 慢性病管理) |
| S3 | S1 OR S2 |
| S4 | (知识图谱 OR 可视化知识图谱 OR 知识图谱可视化) |
| S5 | S3 AND S4  Items found: 106 |
| 8.VIP | |
| S1 | 主题词=慢性病 OR 慢性疾病 OR 慢性非传染性疾病 OR 生活方式病 OR 慢性病管理 |
| S2 | 主题词=知识图谱 OR 可视化知识图谱 OR 知识图谱可视化 |
| S3 | S1 AND S2  Items found: 90 |
